# Ether Spray in Facial Neuralgia

**Published:** 1883-09

**Authors:** 


### Ether Spray in Facial Neuralgia.

Dr. A. M. Cartledge (*Med. Herald*, May, 1883,) protects the eye with a piece of oil silk, and directs a spray of ether upon the part affected until its temperature is down to the freezing point of water—say for eight minutes—which will generally suffice. To generate the spray he uses Richardson's atomizer, which is a simple addition to the ordinary perfume atomizer.

